# Isocyanate-Functionalized Chitin and Chitosan as Gelling Agents of Castor Oil

**DOI:** 10.3390/molecules18066532

**Published:** 2013-06-03

**Authors:** Rocío Gallego, Jesús F. Arteaga, Concepción Valencia, José M. Franco

**Affiliations:** 1 Department of Chemical Engineering, Physical Chemistry and Organic Chemistry, University of Huelva, Campus El Carmen, Campus ceiA3, 21071 Huelva, Spain; 2 CIQSO—Center for Research in Sustainable Chemistry, University of Huelva, 21071 Huelva, Spain; 3 PRO^2^TEC—Chemical Process and Product Technology Research Center, University of Huelva, 21071 Huelva, Spain

**Keywords:** chitin, chitosan, castor oil, isocyanate, lubricating grease, rheology

## Abstract

The main objective of this work was the incorporation of reactive isocyanate groups into chitin and chitosan in order to effectively use the products as reactive thickening agents in castor oil. The resulting gel-like dispersions could be potentially used as biodegradable lubricating greases. Three different NCO–functionalized polymers were obtained: two of them by promoting the reaction of chitosan with 1,6-hexamethylene diisocyanate (HMDI), and the other by using chitin instead of chitosan. These polymers were characterized through ^1^H-NMR, FTIR and thermogravimetric analysis (TGA). Thermal and rheological behaviours of the oleogels prepared by dispersing these polymers in castor oil were studied by means of TGA and small-amplitude oscillatory shear (SAOS) measurements. The evolution and values of the linear viscoelasticity functions with frequency for –NCO–functionalized chitosan- and chitin-based oleogels are quite similar to those found for standard lubricating greases. In relation to long-term stability of these oleogels, no phase separation was observed and the values of viscoelastic functions increase significantly during the first seven days of ageing, and then remain almost constant. TGA analysis showed that the degradation temperature of the resulting oleogels is higher than that found for traditional lubricating greases.

## 1. Introduction

Nowadays, there is a general tendency to promote the replacement of non-renewable raw materials by renewable resources in order to avoid or minimize the impact that process technologies and industrial products cause in the environment. The lubricant industry is especially interested in reducing the environmental impact of its products [1,2]. Although vegetable oils are being increasingly used as lubricant base oils instead of mineral and synthetic oils, the substitution of traditional thickener agents, such as lithium, aluminum, sodium or calcium soaps in lubricating greases by others derived from renewable resources, like some biopolymers, is a complicated task due to the technical efficiency of these metallic soaps to impart the desired functional properties to the final product.

Chitosan is typically obtained by deacetylation under alkaline conditions of chitin, which is the second most abundant biopolymer in Nature, after cellulose. Chitin can be found as an important constituent of the exoskeleton in animals, especially in crustaceans, mollusks and insects, and it is also the principal polymer in the cell wall of certain fungi. Chitin and chitosan are linear polysaccharides composed of randomly distributed β-(1-4)-linked d-glucosamine (deacetylated unit) and *N*-acetyl-d-glucosamine (acetylated unit). Chitosan is available in a variety of useful forms, displays interesting properties such as biocompatibility or biodegradability [3,4] and its degradation products are non-toxic, non-immunogenic and non-carcinogenic [5,6], which make it a very attractive biomaterial. It is extensively used in many different applications such as treatment of wastewater [7], as a chromatographic support [8], in enzyme immobilization [9], as a wound-healing dressing [10], in dental applications [11], in adhesion bandages for surgery [12], and in drug-delivery systems [13,14,15,16].

Nowadays there is a growing interest in the chemical modification of chitosan in order to improve its solubility in different media and widen its applications [17,18,19]. Both chitin and chitosan have two types of reactive groups that can be grafted: the free amine groups on the deacetylated units and the hydroxyl groups on the C3 and C6 carbons on acetylated or deacetylated units. These reactive sites enable the linkage of a wide variety of functional reactive molecules [20,21,22]. One of these molecules that can be linked in those positions is the 1,6-hexamethylenediisocyanate (HMDI), which is commercially available and generally utilized as a strong cross-linker of –NH_2_ or –OH groups, since it possesses two reactive isocyanate groups (–N=C=O) [23]. The –OH groups of chitin and chitosan can react with –NCO groups to form an urethane linkage (–NH–C(O)–O–) due to the transfer of protons from –OH groups to the nitrogen atoms of –NCO units. HMDI can also react with deacetylated amino groups in the biopolymers. The reaction between chitosan and HMDI has been previously promoted to use the resulting products in different applications such as molecular carriers in the biomedical and environmental fields [24], as support material for Pd catalysts used in hydrogenation reactions [25], as solid support for Cu(I) and Pd(II) catalysts used in reactions like azide/alkyne [3+2] cycloaddition and Suzuki cross-couplings [26], cholesterol absorption [23], adsorption of phenols, toxic carcinogens [27], gold sorption [28] or polyurethane elastomers [29].

The use of chitin and chitosan to thicken vegetable oil media has been previously addressed [30], but some limitations regarding physical and mechanical stabilities and rheological response were pointed out as a consequence of a certain degree of chemical incompatibility between oil and such polar biopolymers. Relatively physically stable suspensions were obtained only above the percolation threshold. Besides this, in a previous study [31], the functionalization of methylcellulose with reactive –NCO groups, and its further ability to thicken castor oil by reacting with the –OH groups of the ricinoleic fatty chain was studied. The resulting chemical oleogels may serve for different applications depending on the degree of functionalization. One of these, obtained with methylcellulose functionalized to a lower degree, presented suitable rheological properties and thermal resistance to be proposed as a biodegradable alternative to lubricating greases. Following a similar methodology, the incorporation of reactive –NCO groups into chitin and chitosan and its ability to thicken castor oil is explored in this work in order to avoid the previously mentioned problems and increase the chemical affinity between these polymers and vegetable oils. These oleogels prepared with isocyanate-funcionalized chitin and chitosan polymers and castor oil were rheologically and thermally characterized.

## 2. Results and Discussion

### 2.1. Synthesis and Characterization of Isocyanate—Functionalized Chitin and Chitosan

Three different types of polymers functionalized with isocyanate groups, chitosan in two of the cases and chitin in the other one, were prepared. The introduction of the isocyanate moieties was carried out in order to improve the role of these biopolymers as thickener agents in castor oil. The amount of –NCO groups present in the final polymer will be a determining factor in the ability to act as a suitable thickener. Therefore, two experimental conditions have been selected for the preparation of the modified polymers of chitosan: the first (CSAN-1) used an amount of –NCO moieties (0.5 equiv., Table 1) required for the reaction with half of the deacetylated –OH and –NH groups present in this polymer (1.0 equiv., Table 1); and the second one (CSAN-2) using a lower amount of –NCO (0.25 equiv., Table 1) to promote the reaction with a quarter of the free –OH and –NH groups (1.0 equiv., Table 1). This last level of functionalization was the one chosen for the modification of chitin, thus affording the third isocyanate–functionalized biopolymer (CTIN-1).

**Table 1:** Molar ratios of chitosan, HMDI, Et_3_N and toluene used in the functionalization reaction.

	Starting material	HMDI	Et_3_N	Toluene
	[equiv.] *	[equiv.] *	[equiv.] *	[mL]
CSAN–1	1.00	0.50	1.00	100
CSAN–2	1.00	0.25	0.50	100
CTIN–1	1.00	0.25	0.50	100

* Molar ratio between moles of free –OH and –NH groups present in each monomer of starting chitosan, and moles of each of the reagents employed.

**Figure 1:**
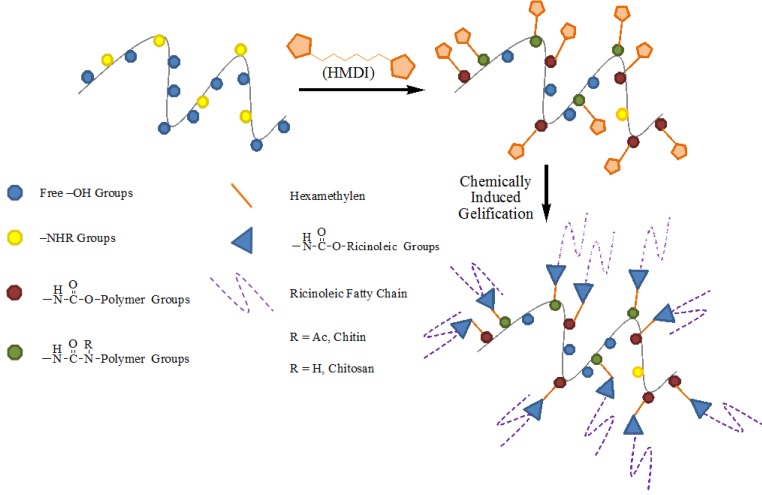
General strategy for chitin and chitosan NCO–functionalization and reaction with ricinoleic fatty acid during oleogel preparation.

The use of two different –NCO/biopolymer ratios allows the control of the functionalization process and obtaining polymers with different number of free –OH and –NCO groups. Because of HMDI’s ability to act as cross–linking reagent, since it possesses a –NCO functional group at each end of the molecule and, therefore, can react once or twice along the course of the experimental procedure, it seems necessary to control these cross–linking reactions to modulate the functional properties of the polymers synthesized. For this reason, the study of the reaction conditions, mainly the use of both reactant ratios described above, was aimed at finding the conditions under which the reaction mainly occurs only at one end of HMDI molecule, which remains linked to the biopolymer, leaving the other free –NCO group able to further interact with the corresponding –OH groups located in the ricinoleic fatty acid chain during oleogel preparation (Figure 1) [31]. Lower quantities of –NCO than that required by the reaction stoichiometric proportions are used to avoid or minimize the possible formation of compounds with a high degree of cross–linking between –NCO groups, namely polyurethanes. The structure of the chemically modified biopolymers, chitin/chitosan–O–NHC(O)–hexamethylene–NCO, contain a wide variety of functional groups (Figure 1) highlighting the presence of urethane linkages between the polymer and hexamethylene–NCO chain responsible for the subsequent reaction with –OH groups of castor oil fatty acids during oleogel preparation. 

**Figure 2:**
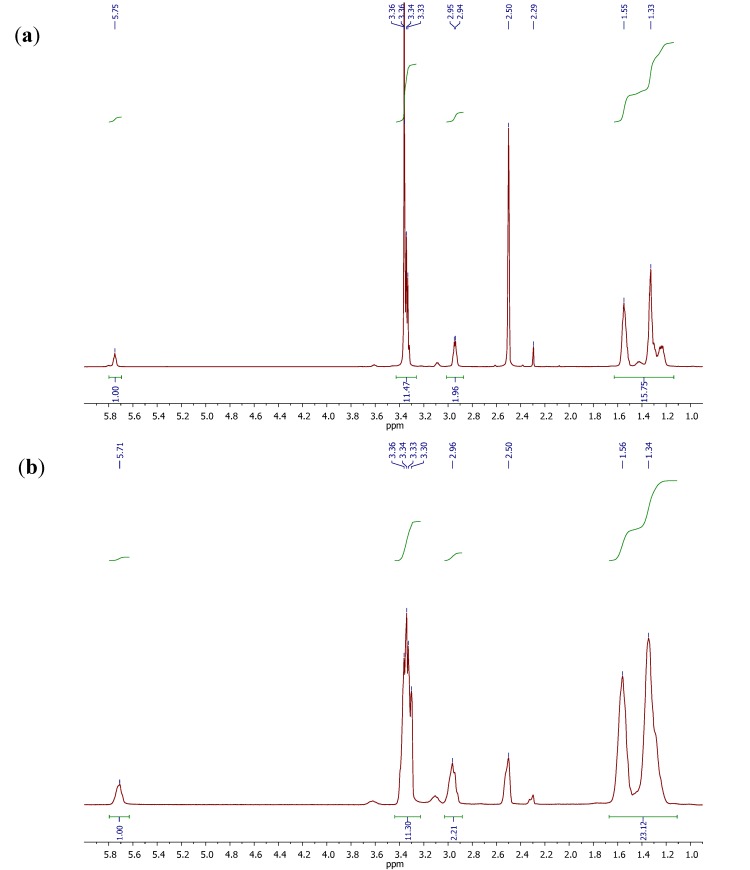
^1^H-NMR spectra for (**a**) CTIN-1 and (**b**) CSAN-1.

Figure 2 shows the NMR spectra of the functionalized chitosan with a higher amount of HMDI (CSAN-1) and isocyanate-functionalized chitin (CTIN-1), respectively. The main signals of both spectra are approximately the same and they are in concordance with the proposed chemical structures. The signals corresponding to the proton of the N–H group at the end of the reacted HMDI chain (5.8 ppm) and the two protons of the –CH_2_ group attached to it (2.9 ppm) are present in a 1:2 ratio, which means that the reaction between chitin or chitosan and the diisocyanate was successfully accomplished. The higher amount of HMDI in CSAN-1 can be noticed by the more intense peaks corresponding to the protons of the four –CH_2_ groups placed in the middle of the HMDI chain (1.6–1.2 ppm). The protons of O–H, N–H, the sugar rings and the –CH_2_ attached to non–reacted HMDI isocyanate groups appear at 3.4 ppm and they are signals corresponding to the main chitosan and chitin structure. The –CH_3_ of the acetylated part displays a peak at 2.3 ppm and the solvent, DMSO-d_6_, shows a noticeable signal at 2.5 ppm.

**Figure 3:**
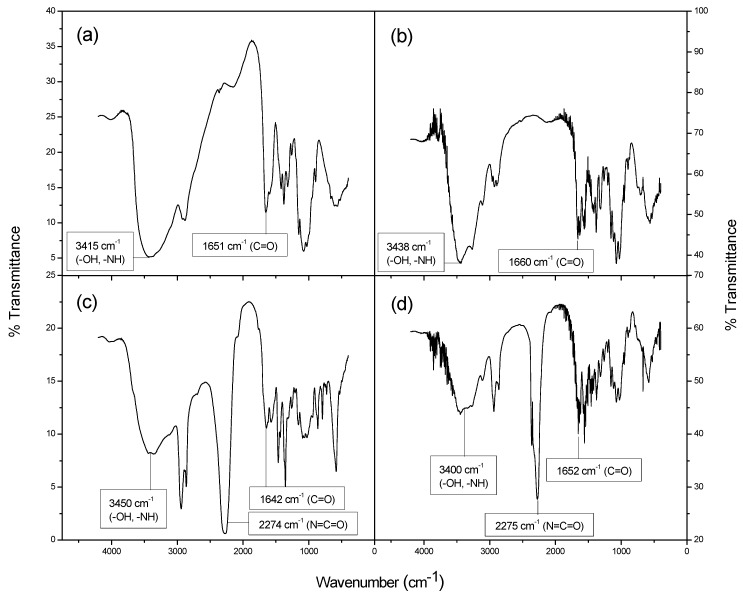
FTIR spectra for (**a**) chitosan, (**b**) chitin and polymers, (**c**) CSAN-2 and (**d**) CTIN-1.

**Figure 4:**
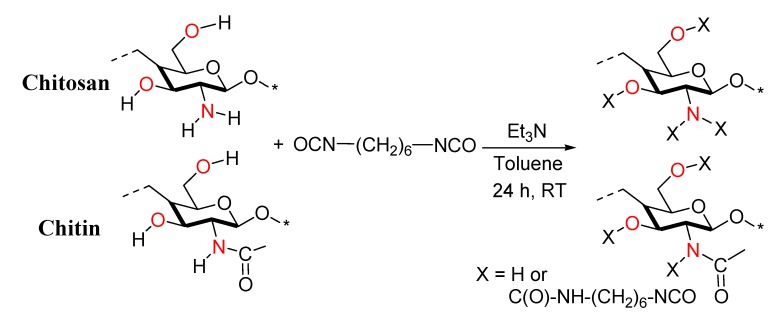
Reaction of chitosan and chitin with hexamethylene diisocyanate (HMDI).

Fourier transform infrared spectroscopy (FTIR) was used to evaluate the chemical modification of chitosan and chitin after the functionalization with HMDI. Figure 3 shows the FTIR spectra of chitosan, chitin and the corresponding samples once functionalized with HMDI (CSAN-2 and CTIN-1, respectively). Infrared spectra of new functionalized polymers were in concordance with the proposed chemical structures. The reduction in intensity of the bands at 3,357 cm^−1^ (CSAN-2) and 3,364 cm^−1^ (CTIN-1) attributable to the –OH moieties in chitosan and chitin, once reacted with isocyanate groups of HMDI leading to urethane linkages (see Figure 4) is significant. Secondly, the urethane bands were apparent at 3,357 cm^−1^ (CSAN-2) and 3,364 cm^−1^ (CTIN-1) for the N–H group, 1,642 cm^−1^ (CSAN-2) and 1,652 cm^−1^ (CTIN-1) for the C=O group of the urethane and 1,572 cm^−1^ (CSAN-2) and 1559 cm^−1^ (CTIN-1) for the N–H group in the urethane linkages of the modified biopolymers. Moreover, the intense peaks at 2,274 cm^−1^ (CSAN-2) and 2,275 cm^−1^ (CTIN-1) confirmed the presence of unreacted isocyanate groups, as intended.

### 2.2. Thermogravimetric Analysis of Isocyanate-Functionalized Chitosan and Chitin

**Figure 5:**
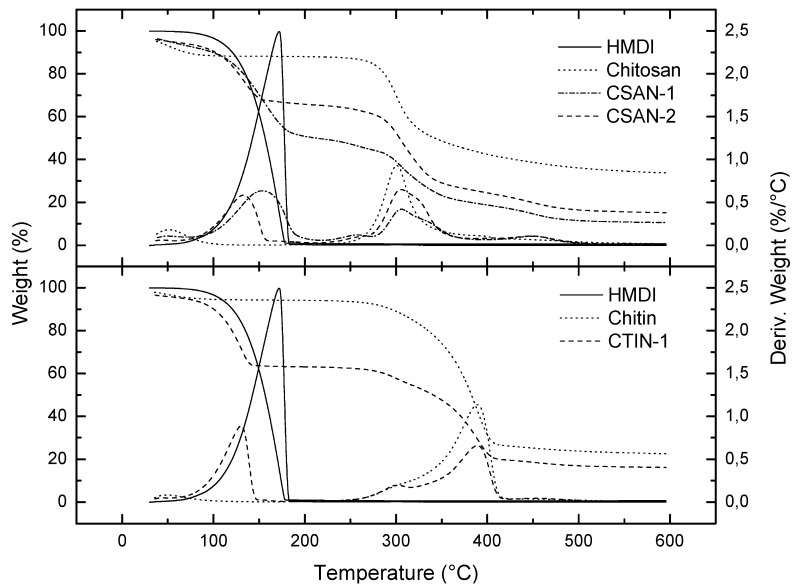
Thermal degradation curves, under inert atmosphere, for NCO–functionalized chitosans and chitins and starting materials.

Thermal decomposition of chitosan and chitin and new –NCO–functionalized chitosan (CSAN-1 and CSAN-2) and chitin compounds (CTIN-1) turned to be quite different (Figure 5). The temperature for the onset of thermal decomposition (T_onset_), the temperature at which decomposition rate is maximum (T_max_), the percentage of non-degraded residue and loss weight at the end of each decomposition step have been calculated from the thermograms of the samples studied (Table 2). For non-functionalized chitosan and chitin, thermal degradation under nitrogen atmosphere occurred in one main step. For chitin, this thermal event occurred in the range of 343–405 °C and was due to the degradation of the saccharide structure of the molecule, including the dehydration of saccharide rings and decomposition of both acetylated and deacetylated chitin units [32]. This decomposition step occurred for chitosan at a lower temperature, indicating lower thermal stability. The peak exhibited at 301 °C corresponds to the degradation of part of the molecule that is deacetylated. This intense peak showed an overlapped small signal at around 390 °C which could correspond to the acetylated part of the molecule. Previously, a certain mass loss, between 43 °C (chitosan)/40 °C (chitin) and 77 °C (chitosan)/79 °C (chitin), must be attributed to water evaporation. For functionalized chitin and chitosan, the inclusion of HMDI segments into the polymer structure reduced their thermal stabilities in great extent due to the –HMDI groups, with marked impact in the highest functionalized polymer, as expected (Figure 5, Table 2).

**Table 2:** TGA characteristic parameters for the biodegradable thickeners studied and starting materials.

Sample	T_onset_	T_max_	T_final_	Residue	*∆W*
	(°C)	(°C)	(°C)	(%)	(%)
Chitosan	43/**281**	51/**301**	77/**472**	34	9/**57**
Chitin	40/**343**	49/**389**	79/**405**	23	4/**72**
HMDI	137	172	178	1	99
CSAN-1	43/**122**/240/**292**/429	50/**153**/258/**306**/450	60/**179**/266/**340**/483	11	4/**44**/6/**26**/9
CSAN-2	40/**107**/**283**/431	43/**133**/**306**/448	50/**150**/**335**/486	15	2/**30**/**43**/9
CTIN-1	**106**/**275**/**363**/439	**130**/**302**/**389**/454	**140**/**309**/**403**/494	16	**34**/**10**/**36**/4

Apart from the loss of water, thermal decomposition of functionalized chitosan polymers exhibited two main stages: the first one takes place between 122 °C (CSAN-1)/107 °C (CSAN-2) and 179 °C (CSAN-1)/133 °C (CSAN-2) due to the loss of –NCO segments; and the second one, between 292 °C (CSAN-1)/289 °C (CSAN-2) and 340 °C (CSAN-1)/335 °C (CSAN-2), is related to the loss of the deacetylated part of the chitosan molecule. Small peaks corresponding to the degradation of the acetylated part can be also detected in the derivative curve at higher temperatures (Figure 2). For the CTIN-1 polymer, three main steps are observed: the first stage takes place from 106 °C to 140 °C and corresponds to the loss of –NCO segments, the second one, between 275 °C and 309 °C, is related to the loss of the deacetylated part of the molecule; and the third stage between 363 °C and 403 °C is due to the loss of the acetylated units. As can be deduced from Table 2, the functionalized chitosan polymer with higher –NCO content (CSAN-1) exhibited higher weight loss in the second stage and lower in the third stage. On the other hand, HMDI thermal degradation also occurs in one single stage, between 137 and 178 °C, which matches the first main degradation stage of functionalized polymers analyzed. This mass loss appears more intensively in CSAN-1 (Figure 5), due to the presence of the higher amount of inserted HMDI.

### 2.3. Thermal and Spectroscopic Characterization of Isocyanate-Functionalized Chitosan and Chitin Gel-Like Dispersions in Castor Oil

**Figure 6:**
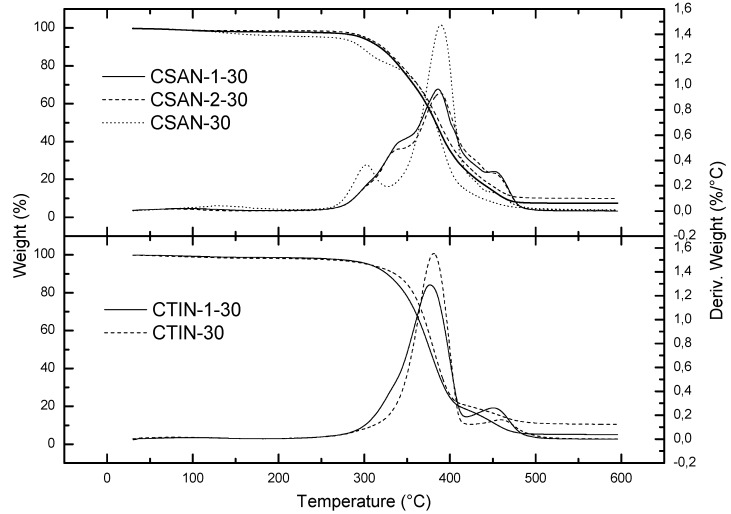
Thermal degradation curves, under inert atmosphere, for –NCO–functionalized and non-functionalized chitosan and chitin gel-like dispersions in castor oil.

Different oleogels were prepared by dispersing 30% w/w of non-functionalized and –NCO–functionalized chitin and chitosan in castor oil. Figure 6 shows that chitosan and chitin derivative-based oleogels exhibit slightly different thermal tendencies. The thermal degradation of non-functionalized chitosan gel-like dispersions (CSAN-30) takes place from 278 °C to 482 °C and three different maximum peaks at 302 °C, 389 °C and 449 °C can be observed (Table 3). The first one corresponds to chitosan degradation and the second is mainly due to castor oil [33]. In the case of oleogels formed with –NCO–functionalized chitosan, the thermal degradation takes place from approximately 297 °C to 481 °C and again three maximum peaks are observed, in this case at 344 °C, 386 °C and 452 °C. The second and the third peaks are similar to those found in the non-functionalized chitosan-based oleogel (CSAN-30), whereas the first peak is delayed. Chitin-based oleogels have two clearly separated degradation steps. The onset temperature for the first one appears close to 342 °C and the final temperature at around 409 °C, with maximum degradation rate at 381 °C, which is attributable to both castor oil decomposition and the main degradation stage of chitin (see Figure 2). The second one takes place from 425 to 595 °C with a maximum peak at 459 °C. A similar degradation pattern was obtained for the –NCO-functionalized chitin-based oleogel. Finally, it can be concluded that the disappearance of the first isocyanate-functionalized polymer degradation peak in the resulting oleogels confirms the chemical reaction promoted between the modified polymer and the castor oil. In general, thermal resistance of these oleogels is significantly higher than those previously reported for traditional lubricating greases [34,35,36].

**Table 3:** Characteristic TGA parameters of the studied oleogel formulations.

Sample	T_onset_	T_max_	T_final_	Residue	*∆W*
	(°C)	(°C)	(°C)	(%)	(%)
CSAN-1-30	297	344/386/452	481	7.40	92
CSAN-2-30	301	339/389/451	481	9.97	90
CTIN-1-30	327/444	376/450	410/485	5.07	95
CSAN-30	278/357	302/389/449	319/482	3.89	96
CTIN-30	342/425	381/459	409/595	10.46	89

**Figure 7:**
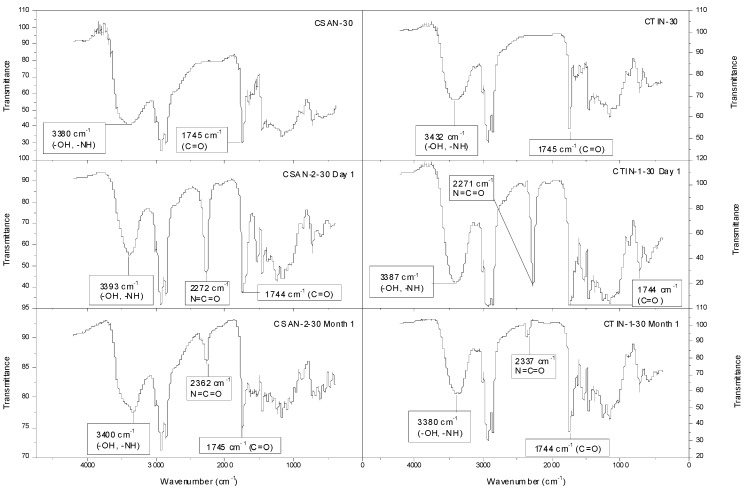
FTIR spectra for selected –NCO-functionalized and non-functionalized chitosan and chitin gel-like dispersions in castor oil.

The reaction between –NCO and –OH groups of polymer and castor oil, respectively, was also studied by analyzing the FTIR spectra. Figure 7 shows the FTIR spectra of four oleogels containing different thickening agents. Chitin- and chitosan-based gel-like dispersions are compared with the corresponding –NCO-functionalized biopolymers-based oleogels, just one day after their preparation and after one month of ageing.

Functionalized chitosan- and chitin-based oleogels show the same behaviour regarding the evolution with ageing (Figure 7). As can be seen, the absorption band assigned to free –NCO, at 2,271 cm^−1^ is quite significant in the IR spectrum of the isocyanate-functionalized polymers-based oleogels one day after the manufacture. On the other hand, the same signal became negligible one month after oleogel preparation, which suggests a slow reaction rate for the promoted reaction. Finally, the spectra of non-functionalized polymer-based oleogels do not present any signal of free –NCO, as expected.

### 2.4. Rheological Characterization of Functionalized Chitin and Chitosan Gel-Like Dispersions in Castor Oil

**Figure 8:**
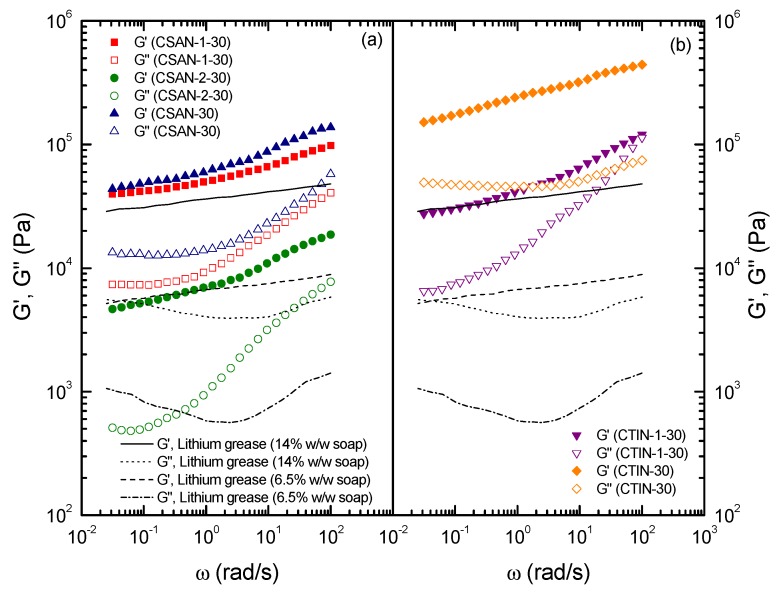
Frequency dependence of the storage [G'] and loss [G''] moduli for NCO–functionalized and non–functionalized (**a**) chitosan and (**b**) chitin gel-like dispersions in castor oil (G': full symbols, G'': open symbols; ageing time: 1 month). Solid and dashed lines correspond to conventional lithium greases containing 14% and 6.5% soap concentration (from reference [41]).

Figure 8 shows the mechanical spectra, obtained from small-amplitude oscillatory shear (SAOS) measurements inside the linear viscoelastic range, for the chitosan- and chitin-based oleogels studied, one month after their preparation. The linear viscoelastic functions for chitin- and chitosan-based oleogels exhibit values of the storage modulus, G', significantly higher than those found for the loss modulus, G'', in the whole frequency studied. In all cases, the so-called ‘‘plateau region’’ was noticed, where G' slightly increases with frequency and G'' displays a minimum. This evolution is typically found in highly entangled polymeric systems [37] and is very similar to that shown by other oleogels previously studied [30,31,34,38,39,40] and standard lubricating greases [41]. Moreover, in the case of the isocyanate-functionalized chitin oleogel (CTIN-1-30) a tendency to achieve a crossover between both linear viscoelastic functions, at high frequencies, can be observed.

As extensively investigated [35,36,42] typical G’ values in lubricating greases of NLGI grade 1–2 range from 10^4^ to 10^5^ Pa, around one order of magnitude higher than G” values, depending on compositions and processing conditions. 

An example of mechanical spectrum for a conventional lithium grease of NLGI grade 2 (14% w/w lithium soap) is included in Figure 8. This particular evolution with frequency and similar values of the SAOS functions were obtained for the –NCO–functionalized chitosan-based oleogel with higher –NCO content (CSAN-1-30) and the non-functionalized chitosan-based gel-like dispersion (CSAN-30). However, the –NCO–functionalized chitosan with lower –NCO content (CSAN-2) produces an oleogel with significantly lower values of the viscoelastic moduli. Therefore, it is clearly noticed that the values of both SAOS functions for –NCO–functionalized chitosan-based oleogels increase with the functionalization degree, as expected attending to the higher degree of cross-linking produced by the higher –NCO content in the biopolymer. In the case of the lower functionalization, the mechanical spectrum is more similar to that exhibited by softer conventional lithium greases with lower soap concentrations (see Figure 8). Nonetheless, in general, the values of G” at high frequencies for the chitosan-based oleogels studied increase more rapidly than found in traditional lithium greases. For chitin-based oleogels, the values of the viscoelastic functions are higher than those required for traditional lubricating greases, although chitin functionalization in a relatively low degree yields an oleogel with significantly reduced values of the SAOS functions. In this sense, NCO–functionalization may be considered a chemical tool to modulate the rheological response of chitin- and chitosan-based oleogels and design formulations comparable to traditional greases of different NLGI number.

These results can be explained attending to the physical mechanism for which these biopolymers are able to thicken castor oil. In fact, non-functionalized chitin and chitosan form physically stable gel-like dispersions in castor oil only above the percolation threshold, at around 30% w/w concentration, showing the typical rheological response of soft solid-like gels [43]. These gel-like characteristics are achieved via intermolecular hydrogen bonding and dipole-dipole interactions involving hydroxyl and amine groups of the chitin and chitosan chains and polarized groups of triglyceride molecules. However, as previously reported for methylcellulose dispersions in vegetable oils [31], below these concentration threshold the dispersions became unstable. On the contrary, the NCO–functionalization procedure induces a real chemical gelation during the biopolymer dispersion process, which allows one to obtain stable oleogels, even at a lower concentrations, also reducing the values of the viscoelastic functions as shown in Figure 8. This gelification process is promoted by the chemical interaction between –NCO functional groups and castor oil –OH groups, as previously demonstrated with FTIR results. Finally, a relatively high degree of HMDI functionalization increases the values of the SAOS functions up to level of those shown by the non-functionalized chitin or chitosan gel-like dispersion, as a consequence of an extensive cross-linking between the reactive biopolymer and the oil medium. Therefore, desired values of linear viscoelastic functions could be selected by controlling the functionalization degree of these biopolymers.

**Figure 9:**
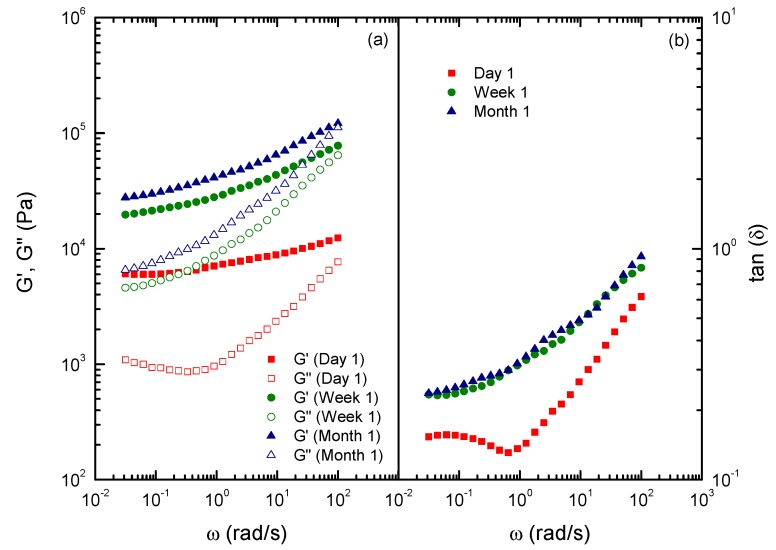
Frequency dependence of the (**a**) storage [G'] and loss [G''] moduli, and the (**b**) loss tangent for the NCO–functionalized chitin gel-like dispersion in castor oil (CTIN-1-30), as a function of the ageing time (G': full symbols, G'': open symbols).

In relation to the evolution of oleogels with time, Figure 9 shows the mechanical spectra for a selected oleogel prepared by dispersing the isocyanate-functionalized chitin in castor oil as a function of the ageing time. As can be noticed, the values of SAOS functions significantly increase during the first seven days of ageing and then slightly increase one month after oleogel preparation, remaining almost constant later on. As previously mentioned, these results can be explained on the basis of the slow reactivity of residual –NCO functional groups in a high viscous medium [44], especially during the first week after oleogel preparation, increasing the degree of cross-linking. Obviously, since no reaction occurs when using the unmodified chitin as thickener, no ageing influence was detected in that case. Moreover, the values of the loss tangent noticeably increase during the first week, resulting almost unaffected afterwards [Figure 9(b)].

## 3. Experimental

### 3.1. Materials

Castor oil (211 cSt at 40 °C, Guinama, Valencia, Spain) was selected as lubricant base oil for oleogel formulations studied. Chitosan (degree of deacetylation = 86.3%, molecular weight = 3.0 × 10^5^ g·mol^−1^) and chitin (degree of deacetylation = 7.3%, molecular weight = 5.4 × 10^5^ g·mol^−1^), purchased from Qingdao Fraken (Qingdao, Shandong, China), were modified with 1,6-hexamethylene diisocyanate (purum grade, ≥98.0%), supplied by Sigma-Aldrich (Saint Louis, MO, USA). All other common reagents and solvents employed were purchased from Sigma-Aldrich.

### 3.2. General Methods

All functionalization reactions (Figure 4) were performed in flasks that were flame-dried after assembly under a positive flow of argon to eliminate surface moisture, and conducted under argon. HMDI was stored at 4 °C and handled under inert (argon) atmosphere. Solvents were purified according to standard literature techniques and stored under argon. Toluene was freshly distilled immediately prior to use from sodium/benzophenone and strictly deoxygenated for 30 min under argon. Reagents were purchased at the higher commercial quality and used without further purification, unless otherwise stated. Characterization tests were performed at least in duplicate.

### 3.3. Functionalization Reaction of Chitosan and Chitin with HMDI

The reactions of chitosan and chitin functionalization (Figure 4) were carried out following the methodology previously reported [31]. In the case of chitosan, the amount of HMDI, Et_3_N and toluene used was modified with the aim of controlling the ratio of functionalized –OH and –NH_2_ groups in the chitosan derivative synthesized. The different ratios of HMDI, Et_3_N and toluene with respect to starting material used in the reaction are listed in Table 1.

#### 3.3.1. CSAN-1 Synthesis

Chitosan (10.0 g, 62.0 mmol) was added to a bottom round flask with toluene (100.0 mL) while stirring at room temperature becoming a suspension. Then Et_3_N (35.0 mL, 250.2 mmol) and HMDI (20.0 mL, 122.4 mmol) were also added to the system, the last one dropwisely. The solution was vigorously stirred at room temperature during 24 h. The synthesis was carried out under inert atmosphere of argon. Afterwards, the mixture was vacuum filtered resulting in 35.9 g of a pale yellow powder in a yield of 87%. The product was not stored but rather used immediately.

#### 3.3.2. CSAN-2 Synthesis

CSAN-2 was synthesized following the just described procedure above, but modifying the quantities of reagents. Chitosan (10. 0 g, 62.0 mmol) was added to toluene (100.0 mL) and then Et_3_N (17.0 mL, 121.5 mmol) and HMDI (10.0 mL, 61.2 mmol) were also added and stirred for 24 h. The obtained product was dried under vacuum leading to the polymer CSAN-2 (21.4 g, 52% yield).

#### 3.3.3. CTIN-1 Synthesis

CTIN-1 was synthesized following the same procedure as for polymers CSAN-1 and CSAN-2, but using chitin as starting material and modifying the quantities of reagents. Chitin (12.0 g, 59.1 mmol) was added to toluene (100.0 mL) and then Et_3_N (12.4 mL, 88.6 mmol) and HMDI (7.2 mL, 44.0 mmol) were also added and stirred for 24 h. The obtained crude was dried under vacuum leading to 22.4 g of product in a yield of 70%.

### 3.4. Preparation of Oleogels

Oleogel samples were prepared in an open vessel, using a controlled-rotational speed mixing device (70 rpm) RW 20 (Ika), equipped with an anchor impeller to disperse the different biopolymers in castor oil. Functionalized biopolymers were slowly added to the oil at a concentration of 30% w/w. The mixing process was maintained at 70 rpm for 24 h at room temperature. Finally, the resulting dispersion was homogenized with an Ultra-Turrax T50 (Ika) rotor-stator turbine, at 8800 rpm during 1 min. Batches of 60 g were prepared for each formulation investigated (see Table 4).

**Table 4:** Oleogels studied.

Thickener agent	Code applied
CSAN-1	CSAN-1-30
CSAN-2	CSAN-2-30
Chitosan	CSAN-30
CTIN-1	CTIN-1-30
Chitin	CTIN-30

### 3.5. Thermogravimetric Analysis (TGA)

Measurements of mass losses *versus* temperature were carried out by using a Thermogravimetric analyzer, model Q-50 (TA Instrument Waters, New Castle, DE, USA) under N_2_ purge. Typically, 5–10 mg of sample were placed on a platinum pan and heated from 30 °C to 600 °C, at 10 °C/min.

### 3.6. Nuclear Magnetic Resonance of Protons (^1^H-NMR)

NMR spectra were recorded with a Varian Direct-Drive 500 (^1^H 500 MHz) spectrometer using DMSO-d_6_ as solvent.

### 3.7. Fourier Transform Infrared Spectroscopy (FTIR)

FTIR spectra were obtained with a Digilab FTS3500ARX (Varian) apparatus. Biopolymer samples were prepared as KBr pellets, whereas a small drop of oleogels was placed between two KBr disks (32 × 3 mm). Then, in both cases, the set was placed into an appropriate sample holder. The spectra were obtained in a wavenumber range of 400–4,000 cm^−1^, at 4 cm^−1^ resolution, in the transmission mode.

### 3.8. Rheological Characterization

Rheological characterization of oleogels was carried out with a Gemini controlled-stress rheometer (Malvern, Worcestershire, UK). Small-amplitude oscillatory shear (SAOS) tests were performed inside the linear viscoelastic region, using plate-plate geometries (25 mm, and 1 mm gap), in a frequency range of 10^−2^–10^2^ rad/s, at 25 °C. At least two replicates of each test were performed on fresh samples, one day, one week and one month after oleogel preparation. Average standard deviation for G' and G'' moduli was always lower than 4.5%.

## 4. Conclusions

Three new biopolymers were successfully synthesized by inducing the functionalization reaction of chitosan and chitin with 1,6-hexamethylene diisocyanate (HMDI). The introduction of the isocyanate moieties was carried out in order to improve the role of these polymers as thickener agents in a castor oil medium for lubricant applications. Two experimental conditions have been selected for the preparation of the modified polymers of chitosan: the first one using a higher amount of –NCO (0.5 equiv.) and a second one using a lower quantity (0.25 equiv.) in order to control the functionalization process and obtain polymers with different numbers of free –OH groups. Afterwards, several gel-like dispersions were prepared and rheologically characterized by dispersing these isocyanate-functionalized biopolymers in castor oil. The linear viscoelastic responses in SAOS tests of some –NCO-functionalized chitosan- and chitin-based oleogels are quite similar to those found for standard lubricating greases. The values of viscoelastic functions increase during one month of ageing, more noticeably during the first seven days, but the relative elasticity of the systems is almost unaffected after one week. From the thermogravimetry point of view, in general, thermal decomposition of functionalized biopolymers-based oleogels takes place practically in one main single stage which involves the thermal degradation of both biopolymer and castor oil, being the thermal resistance higher than that found for traditional lubricating greases. In conclusion, –NCO-functionalization of chitin and chitosan allows one to obtain more stable and homogeneous oleogels, at the same time that it serves as a powerful chemical tool to modulate the rheological response and design formulations comparable to traditional greases of different consistency.
